# The effect of a cluster randomised control trial on objectively measured sedentary time and parental reports of time spent in sedentary activities in Belgian preschoolers: the ToyBox-study

**DOI:** 10.1186/s12966-015-0325-y

**Published:** 2016-01-05

**Authors:** Marieke De Craemer, Ellen De Decker, Maïté Verloigne, Ilse De Bourdeaudhuij, Yannis Manios, Greet Cardon, on behalf of the ToyBox-study group

**Affiliations:** Department of Movement and Sport Sciences, Ghent University, Watersportlaan 2, Ghent, 9000 Belgium; Research Foundation Flanders, Brussels, Belgium; Department of Nutrition and Dietetics, Harokopio University, E. Venizelou 70, Athens, 17671 Greece

**Keywords:** Sedentary behaviour, Screen time, Intervention, Preschoolers, Randomized cluster trial

## Abstract

**Background:**

In preschoolers, high levels of sedentary behaviour are associated with several adverse health outcomes. The purpose of this study is to report the effects of the ToyBox-intervention (a European 24-week cluster randomised controlled trial) on sedentary behaviour in preschoolers.

**Methods:**

In Belgium, 859 preschoolers from 27 kindergartens (15 intervention and 12 control) wore an accelerometer to objectively measure their sedentary time and 1715 parents/caregivers completed a questionnaire to assess sedentary activities in which preschoolers participate at home. Main outcomes were objectively measured sedentary time, time spent watching TV, using the computer and time spent in quiet play. Multilevel repeated measures analyses were conducted to take clustering into account. Intention to treat analysis was used to handle missing data.

**Results:**

A sample of 859 (29.5 % of all contacted children) preschoolers (4.4 ± 0.6 years, 54.4 % boys) provided valid accelerometer data at either baseline or follow-up and parents of 1715 (58.9 % of all contacted children) preschoolers (4.4 ± 0.5 years, 52.5 % boys) completed a questionnaire at either baseline or follow-up. No intervention effects were found on objectively and subjectively measured total sedentary time in the total sample. However, some effects on objectively and subjectively measured sedentary time were found in specific subgroups. Preschoolers from the intervention group from high SES kindergartens and preschoolers with high levels of sedentary time at baseline decreased their sedentary time, while preschoolers from the control group increased their sedentary time. Girls in the intervention group decreased their TV viewing time during weekend days (-5.83 min/day), while girls’ &TV viewing in the control group increased (+4.15 min/day). In low SES kindergartens, a smaller increase for computer time during weekend days was found in preschoolers in intervention kindergartens (+6.06 min/day) than in control kindergartens (+12.49 min/day).

**Conclusion:**

While some small positive effects were found in some sub-groups, the ToyBox-intervention had no effect on objectively and subjectively measured sedentary time in the total sample. A longer period to implement the intervention and a more active involvement of parents/caregivers might enhance intervention effects.

The ToyBox-study is registered with the clinical trials registry clinicaltrials.gov, ID: NCT02116296.

## Background

Preschool children spend high proportions (50–80 %) of their waking time in sedentary behaviour [1–5]. Research has demonstrated that sedentary behaviour is not the opposite of physical activity. These behaviours are two unique behavioural constructs and are independently related to health outcomes [6]. High levels of sedentary behaviour (and in particular screen viewing behaviours) moderately track from early childhood to later childhood [1, 7] and these behaviours are also likely to track into adulthood [8, 9] and are related to adverse health outcomes [10–13]. For example, high levels of sedentary behaviour contribute to the imbalance in different energy balance-related behaviours (EBRB’s) (i.e., physical activity, dietary intake, sedentary behaviour) and are associated with excessive weight gain in young children [14]. For that reason, sedentary behaviour guidelines for preschoolers have been established that suggest to limit prolonged periods of sitting [15–17]. Sedentary behaviour guidelines for preschoolers also include specific recommendations for the amount of screen time per day, as screen time is the most common sedentary behaviour in preschoolers, and is therefore frequently used as a proxy marker of overall sedentary behaviour [18, 19]. These guidelines recommend that preschool children should limit watching television (TV) and the use of other electronic media – like computer, DVDs and other electronic games – to less than one hour per day [15]. Some studies showed that only a small percentage of preschool children adhere to these guidelines. For example, in a Canadian cross-sectional study, 17.9 % of three- to four-year-old preschool children complied with the screen time guideline of spending less than one hour of screen time per day [20]. Similar results were found in an Australian sample of preschoolers (mean age of 4.5 years), with 21.8 % of preschool children adhering to these screen time guidelines [21].

Because of the negative health outcomes related to sedentary behaviour, reducing children’s sedentary time is suggested to be included in health promotion interventions [22]. Different reviews summarized the effects of interventions that focused on this behaviour in children and reported small intervention effects [22–28]. Because preschoolers often have easy access to televisions (TVs), intervention studies have focused on this context of preschoolers’ sedentary behaviour to decrease this type of sedentary behaviour. The school-based intervention study by Dennison et al. (2004) in 2- to 5-year-old US preschoolers implemented seven educational sessions in which suggestions for alternative activities to watch TV were made; for example, ‘no TV signs’ were crafted and stories were read [29]. After the intervention period, TV and video viewing time of preschoolers in the intervention group decreased by 3.1 hours per week, compared to the control group in which children increased their TV viewing time by 1.6 hours per week. Another intervention study executed by Epstein et al. (2008) was implemented in the home environment of 4- to 7-year-old US preschoolers and used the ‘TV Allowance device’, to control and monitor the use of screen viewing devices at home [30]. After the intervention period, both groups decreased their TV viewing time, but preschoolers from the intervention group showed a stronger decrease (17.5 hours per week) in screen time compared to preschoolers from the control group (5.2 hours per week). These two effective intervention studies only focused on decreasing preschoolers’ screen viewing time and did not include other sedentary activities in which preschoolers often engage (e.g., quiet play, looking at books, passive transportation). However, it is recommended to focus on different forms of sedentary behaviour (i.e., screen viewing activities and non-screen viewing activities), because it is more likely that a decrease of time spent in one sedentary behaviour will be allocated to other sedentary activities, rather than in light or moderate physical activity [22].

The ToyBox-study applied a multi-factorial evidence-based approach that used behavioural models in understanding and promoting fun, healthy food, play and policy for the prevention of overweight in early childhood and has developed a theory and evidence-based kindergarten intervention with family involvement [31]. The main purpose of the ToyBox-intervention was to change four different EBRB’s (water consumption, healthy snacking, physical activity and sedentary behaviour) in relation to the prevention of overweight in preschool children aged 4- to 6-years-old [31]. Different forms of preschoolers’ sedentary behaviour, namely a decrease of preschoolers’ total sedentary time, screen time and quiet play (e.g., looking at books, playing with blocks, making puzzles, drawing) were targeted in the sedentary behaviour module [32]. As overall sedentary time does not consist of screen time alone, it is important to measure preschoolers’ time spent in quiet play as well, since quiet play is categorised as productive sedentary behaviour [19]. Because socio-ecological models point out that health behaviours are influenced by different factors (i.e., personal factors, environmental factors) [33–35], these behaviours can interact at multiple levels such as at the home or the school environment. Therefore, a multi-level approach was chosen. Since a large population of preschoolers across ethnic and socio-economic groups can be reached in kindergartens or child-care settings [36], these settings were used for the implementation of this health promotion intervention. Apart from teachers in the kindergarten environment, also parents/caregivers in the home environment participated in this intervention, as parents/caregivers have an important influence on children’s behaviours in early childhood [37].

The present study aimed at evaluating the effects of the ToyBox-intervention module focusing on decreasing preschoolers’ sedentary behaviour in Belgian preschoolers. The first aim of this study was to examine the effect on preschoolers’ objectively measured total sedentary time, separately examined for weekdays, weekend days, during and after school hours. Furthermore, we also wanted to examine the effect of the intervention on different types of sedentary activities. The second aim of this study was therefore to examine the effect of the intervention on preschoolers’ screen time and the time they spent in quiet play. Because an association between sedentary behaviour and child’s gender [38–41] and parental education as an indicator of socio-economic status (SES) was found in previous studies [42, 43], the third aim of our study was to investigate if possible intervention effects differed according to child’s gender and kindergartens’ SES. Finally, we also examined the intervention effects in a sub-group of preschoolers who had the highest levels of sedentary time at baseline.

## Methods

### Study protocol and subjects

The ToyBox-intervention was implemented in a randomized controlled cluster intervention with a pre-test post-test design including kindergartens or kindergarten centres in an intervention and control condition in six European countries (Belgium, Bulgaria, Germany, Greece, Spain, and Poland) (www.toybox-study.eu). Preschoolers and their families across these countries were recruited at kindergartens, day-care centres or preschool settings, depending on the country regulations and legislation. In order to avoid confusion for the reader, the settings in which Belgian preschoolers were recruited will be referred to as “kindergartens” throughout this paper. Belgian preschoolers can attend kindergarten from 2.5 to 6 years old. It is not compulsory but about 98 % of preschool-aged children in Belgium attend kindergarten [44]. Kindergartens run from Mondays until Fridays and preschoolers spend approximately 30 hours per week at kindergarten [45]. In Belgian kindergartens, teachers pursue the developmental goals in terms of knowledge, insight, skills and attitudes that are imposed by the government [46]. Accelerometers to objectively measure preschoolers’ sedentary time were only used in one country (Belgium), which is why only the Belgian data were used in the present study.

As described in the standardized protocol of the ToyBox-study, tertiles of municipalities in two provinces (East- and West-Flanders) in Flanders, the northern part of Belgium, were created for the implementation of the intervention. First, all municipalities in those two provinces were listed and SES levels (i.e., low, medium and high SES) of each municipality was determined based on the educational level of the respective residents which was collected through the National Statistical Service [47]. A random sample of municipalities from each tertile with low, medium or high SES was taken and kindergartens located in those selected municipalities were contacted. A total sample of 97 kindergartens with a low, medium or high SES, based on the SES level of the municipality and located in this municipality, were invited to participate in the study. The project was first explained to the kindergartens’ principals by phone. If kindergartens requested, a personal visit was performed in order to provide the kindergarten staff with more details about the ToyBox-study. Twenty-seven kindergartens (27.8 %) agreed to participate. The main reason for kindergartens not to take part in the study was the fact that they did not want to overload their teachers. All preschoolers of the first (i.e., three-year-olds) and second (i.e., four-year-olds) kindergarten class (including preschoolers born in January 2007 and December 2008) received an information letter to take home in which the purpose of the study was explained to the parents. In this information letter, parents were invited to participate in the project, to complete a questionnaire with questions regarding preschoolers’ sedentary behaviour and to let their child wear an accelerometer for six consecutive days. After the recruitment of the kindergartens had finished, each municipality was randomly drawn to the intervention or the control condition (2:1) by the project coordinator (Greece) with the use of a command in Excel, which means that the randomisation occurred automatically and electronically. The teachers who were allocated to the control condition kindergartens did not participate in the training session or received the intervention materials. They were informed that they would receive the intervention package after the follow-up measurements and were asked to continue with their normal curriculum during the school year 2012–2013.

Power analyses were performed before the start of the study using the software http://www.statisticalsolutions.net, and were based on a previous kindergarten-based intervention study [48]. As the main outcome of the ToyBox-intervention was preschoolers’ body mass index (BMI), differences in BMI were used in the power analyses. Based on current literature [49], a baseline value for preschoolers’ BMI was 16.35 kg/m^2^, an expected follow-up value of 16.17 kg/m^2^, a standard deviation of 1.73, an α-value of 0.05 and a power of 0.80 were used, resulting in a minimum sample of 726 preschool children which should be achieved. For this reason, at least 800 preschool children with complete data at baseline and follow-up per intervention country were aimed for. To account for potential drop-out, each country had to recruit a sample of minimum 1100 preschoolers [50].

A baseline measurement period (before the implementation of the intervention) and a follow-up measurement period were prescribed in the project protocol. Baseline collection started on schooldays in March 2012 and lasted until the end of June 2012, while follow-up data collection occurred one year later between March and June 2013 to account for possible seasonal effects. Baseline measurements (March-June 2012) took place before summer holidays (July-August 2012) and before the intervention (September 2012-March 2013). Follow-up measurements took place during the last month of the intervention and after the intervention (March-June 2013). During both these measurement periods, the study outcomes were collected in both the intervention and control kindergartens. Children with written informed consent to wear an accelerometer were fitted with the device to obtain objectively measured data on their sedentary time. Furthermore, all preschoolers who participated in the project received a questionnaire to take home and parents were invited to fill in this questionnaire. At follow-up, parents received the same questionnaire and accelerometer data from the same preschoolers were collected. The ToyBox-study was approved by the Ethics Committee of the University Hospital of Ghent (EC/2010/037). Furthermore, the ToyBox-study is registered with the clinical trials registry clinicaltrials.gov, ID: NCT02116296.

### The sedentary behaviour module of the ToyBox-intervention

The structured planning and development of the ToyBox-intervention, including the sedentary behaviour module, was done using the Intervention Mapping Protocol [51]. A detailed description of the development of the sedentary behaviour module in the ToyBox-intervention can be found elsewhere [32]. This kindergarten-based family-involved intervention was implemented by the kindergarten teachers and before the intervention started, two one-hour teachers’ trainings per kindergarten were conducted. The first training session was conducted before the start of school year 2012-2013 to inform teachers about environmental changes they could perform in their classroom. In the second training session, which was performed in September 2012, the sedentary behaviour intervention was explained to the teachers and teachers were also provided with all the intervention material. Before the start of the repetition period (which will be explained below), teachers received a third teacher training session.

**Fig. 1:**
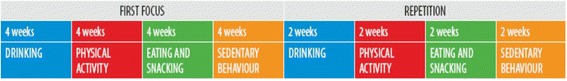
Visual representation of the process of the ToyBox-intervention

The entire ToyBox-intervention lasted from September 2012 until March 2013 for 24 weeks in the school year 2012–2013, with the sedentary behaviour module implemented in weeks 13 until 17 and a repetition period in weeks 23 and 24 (Fig. 1). During each intervention module, teachers were asked to use the ToyBox-material for at least one hour per week and performed several activities that were listed in a classroom activity guide. The classroom activity guide for sedentary behaviour included three parts and a detailed description of different activities that could be done at kindergarten to decrease and interrupt preschoolers’ sedentary time. The first part of the teachers’ guide included the suggestion of environmental changes in the classroom to decrease sedentary behaviour (e.g., put computers on a raised desk). The environmental changes were applied for 24 weeks. In the second part of the guide, long and short movement breaks that should be performed twice in the morning and twice in the afternoon were suggested. Different stories about a kangaroo and its friends who want to change their sedentary behaviour were also included in the activity guide and could be read to the preschoolers. Furthermore, fun activities to decrease preschoolers’ screen time (e.g., the creation of a week calendar on which preschoolers could put stickers on days they did not watch TV) and quiet play (e.g., playing with blocks while standing) were provided in the last part of the classroom activity guide. The execution of the first two parts of the classroom activity guide continued until the end of the school year (Table 1).

**Table 1:** Details about the ToyBox-intervention materials for the sedentary behaviour module

Material	User	Content	Implementation
Classroom Activities Guide	Teachers	Part 1:	
		Environmental changes in the classroom:	Throughout whole school year
		- Standing play stations - Doing activities while standing - Use the hallway	
		Part 2:	
		Child performing the actual behaviour; i.e. short movement breaks:	Throughout whole school year
		- Spin the wheel - Play statue - Flamingo -Marching - Fireworks - …	During the first focus (weeks 13–16) and repetition period (weeks 23–24)
		Part 3:	
		Classroom activities:	
		- Kangaroo stories - Longer movement breaks - Movement corners - Television bingo - “No TV”-signs - Weekly calendar - …	
Kangaroo hand puppet	Teachers	The kangaroo is used to support the activities that are being carried out. The kangaroo is a mascot of the study.	Throughout whole school year
Newsletters	Parents	Newsletter 1:	
		- General information about sedentary behaviour - Guidelines regarding screen time and sedentary behaviour - Tips to limit children’s time spent sedentary - Activities that are being carried out at kindergarten - Tips for movement breaks	During the first focus (weeks 13–16)
		Newsletter 2:	
		- Guidelines regarding screen time - Tips to decrease children’s screen time - Activities that are being carried out at kindergarten - Parents are a role model	
			During the repetition period (weeks 23–24)
Tip-cards	Parents	Tip-card 1:	
		- Tips to replace sedentary behaviour into active behaviour - Tips on how to motivate the child	During the first focus (weeks 13–16)
		Tip-card 2:	
		- Tips on how to decrease screen-related activities - Tips for parent-child activities	During the repetition period (weeks 23–24)
Poster	Parents	Key messages:	
		- Don’t sit down for a long time – get up and be active! - Do not eat in front of screens! - Limit screen viewing activities – make your own experiences! - Include active movement breaks in the children’s daily lives!	During the first focus (weeks 13–16)

During the implementation of the sedentary behaviour module in the kindergartens, parents/caregivers received two newsletters and two tip-cards, containing different tips and strategies to decrease preschoolers’ sedentary behaviour and screen time at home. In these newsletters, also the recommendation to limit screen time to less than one hour per day was provided. Finally, a poster including key messages to decrease sedentary behaviour was also handed out by the teachers to the preschoolers to take home.

Process evaluation tools were developed to gain insight into the role of the main implementers and their fidelity in implementing the ToyBox-intervention (i.e., teachers and parents/caregivers). Teachers received monthly logbooks, containing questions on changes made to the kindergarten environment, preschoolers’ performing movement breaks, execution of classroom activities, whether they handed out the intervention materials and what their feedback was on the intervention materials. At the end of the ToyBox-intervention, preschoolers’ parents/caregivers received a questionnaire containing questions on whether they received and read the newsletters, tip-cards and poster, and how they perceived these materials (e.g., reliable, understandable, useful).

### Measurement of sedentary time

Three models of ActiGraph accelerometer monitors were used to objectively assess preschoolers’ sedentary time, namely the GT1M (3.8 cm × 3.7 cm × 1.8 cm; 27 g), the GT3X (3.8 cm × 3.7 cm × 1.8 cm; 27 g) and the GT3X+ (4.6 cm × 3.3 cm × 1.5 cm; 19 g). The use of three different accelerometer models was inevitable as all available accelerometers had to be used in order to measure a large sample in a limited amount of time. Only the vertical axis output was used in the present study. There is a strong agreement between the GT1M, GT3X and GT3X+ accelerometer, which makes it acceptable to use these activity monitors together in one study [52].

Before the accelerometers were fitted on the preschoolers, they were initialized using the Actilife Lite software, version 6.5.4 with an epoch measurement interval of 15 seconds [53]. The preschoolers wore the accelerometer on the right hip, secured with an elastic belt around the waist for six consecutive days, including two weekend days. A manual on how to attach the accelerometer was provided to the parents and they were instructed to remove the accelerometer only for sleeping and during water-based activities. Meterplus software, version 4.3 was used to score and clean the accelerometer data assessed during the measurement days (Meterplus, Santech, Inc). The first and the last measurement day were deleted because these days were incomplete. Children were included in the final dataset if they had at least two weekdays and one weekend day with valid data [54]. Non-wearing time was calculated as periods of more than 10 minutes of consecutive zero counts and a valid day was considered when the accelerometer was worn for a minimum of 6 hours per day [53, 55]. Minutes of sedentary time were afterwards estimated using the cut-points from Evenson et al. [56], with ≤ 100 counts per minute categorized as sedentary time as this cut-point is suggested by different studies to provide a good estimate of free-living sedentary time [57, 58].

### Measurement of screen time and quiet play

A Primary Caregivers Questionnaire (PCQ) was developed to assess preschoolers’ sociodemographic factors, and to assess information about parents’ and preschoolers’ physical activity, sedentary behaviour and dietary behaviour. The development of the PCQ was based on previously developed questionnaires from large European studies such as IDEFICS [59], HELENA [60] and ENERGY [61]. More information about the development of the PCQ can be found elsewhere [62]. Parents completed the PCQ both at baseline and follow-up, and questions regarding preschoolers’ TV viewing time (*“About how many hours a day does your child usually watch television (including DVDs and videos) in his/her free time?”*; ICC_week_ = 0.67; ICC_weekend_ = 0.67), computer use (*“About how many hours a day does your child use the computer for activities like playing games on a computer, game consoles (e.g., Playstation, Xbox, GameCube) during leisure time?”*; ICC_week_ = 0.72; ICC_weekend_ = 0.81) and quiet play (*“About how many hours a day does your child have quiet play ((looking at books, playing with blocks, playing with dolls, drawing, construction) during leisure time?”*; ICC_week_ = 0.42; ICC_weekend_ = 0.50) were included in the data analyses [62]. Preschoolers’ time spent in these activities during their leisure time on weekdays and weekend days was assessed by asking parents to indicate how many hours a day their child watched TV/DVDs/videos, how many hours their child used the computer, and how many hours they spent in quiet play with answer possibilities ranging from never, <30 min, 30 min-1 hour/day, 1–2 hours/day, 3–4 hours/day, 5–6 hours/day, 7–8 hours/day, 8 hours/day to > 8 hours per day. Overall, the PCQ showed moderate test-retest reliability for these questions [62]. Test-retest reliability of the PCQ was examined by letting 93 parents from the intervention countries (i.e., Belgium, Bulgaria, Germany, Greece, Poland, and Spain) complete the PCQ twice with a two-week interval [62].

### Anthropometric measurements

Trained researchers visited preschool children at kindergartens and measured their body weight and height according to standardized protocols [63]. Body weight was measured with the SECA 861 calibrated electronic scale (accuracy of 0.1 kg), and body height was measured with the SECA 225 Leicester Portable stadiometer (accuracy of 0.1 cm). Two readings of each measurement were obtained and the mean was used for the analyses. When the two readings differed by more than 1 %, a third measurement was conducted and the mean of the two least deferring values was used. Body Mass Index was calculated as weight/height^2^ (kg/m^2^). Weight status (underweight, normal weight, overweight, obese) was obtained based on the International Obesity Task Force thresholds [64].

### Statistical analyses

For objectively measured sedentary time (primary outcome), all outcomes were separately calculated for weekdays, weekend days, and during school (between 8 AM and 4 PM) and after school hours (between 4 PM and 8 PM). All outcomes were expressed in percentages of the total wearing time by dividing all outcome variables by the total wearing time and multiplying by 100. For measurements of screen time (i.e. TV viewing and computer use) and quiet play (primary outcomes), all variables were recoded into minutes per day using the midpoint method [65] to ensure that numerical outcomes could be used to investigate possible intervention effects. Intention to treat (ITT) analysis was carried out to handle missing data, which means that preschoolers with minimum one valid accelerometer measurement (either at baseline or follow-up), and parents who minimally filled in one complete PCQ (either at baseline or follow-up) were included in the analyses. To impute the missing data, we used the “last observation carried forward” method. Prior to all analyses, all outcome measures were first checked for normal distribution (skewness < 0.70) and appeared to be normally distributed. Descriptive statistics were computed to describe sample characteristics as percentage or means and standard deviations.

To investigate possible changes in objectively measured sedentary time, screen time and quiet play according to the condition, multilevel repeated measures analyses were performed using MLwiN 2.28 (Centre for Multilevel Modelling, University of Bristol, UK). To indicate potential interaction effects according to child’s gender and municipality based SES-level of the kindergarten (secondary outcomes), a three-way interaction effect (time*condition*gender and time*condition*SES) was investigated for each outcome. Furthermore, also a three-way interaction effect (time*condition*group) was investigated for a sub-group of preschoolers who had the highest levels of sedentary time at baseline (i.e., total sedentary time above the 70^th^ percentile at baseline). In case of a significant three-way interaction effect for an outcome variable, analyses were stratified by gender, SES or group. To ensure that clustering of two measurements of children in different classes in the different kindergartens was taken into account, four levels were defined for the multilevel modelling (measurement – child – class – kindergarten). In the total sample and in the subgroups regarding SES and high levels of sedentary time at baseline, we adjusted for gender and age. In the subgroup regarding gender, we only could adjust for age. Two different β-values were calculated during the analyses: (1) the β for the ‘time effect’ is the amount of change in the three outcomes (objectively measured sedentary time, screen time and quiet play) associated with going from pre-test to follow-up, (2) the β-value for the ‘interaction effect’ (time*condition) indicated the difference in the change in objectively measured sedentary time, screen time and quiet play going from pre-test to follow-up according to the condition (intervention vs. control). To indicate the effect size of the significant interaction effects, Cohen’s d statistic values are reported in the text (small effect = 0.20, moderate effect = 0.50, large effect = 0.80) [66]. Statistical significance level was set at *p* < 0.05 to account for multiple testing. Furthermore, the 95 % confidence intervals were calculated as well.

## Results

**Fig. 2:**
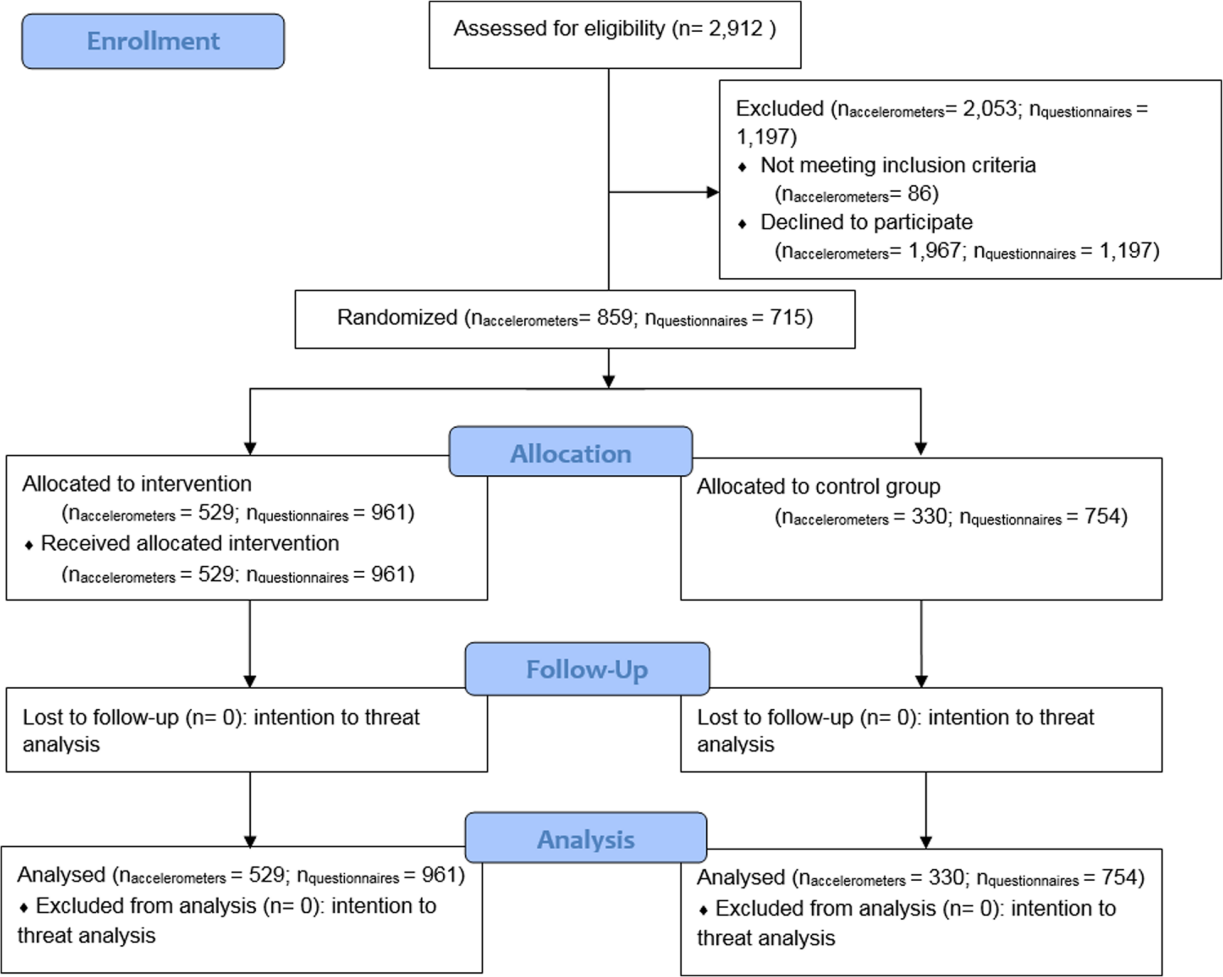
Flow chart of included kindergartens and preschoolers into the Belgian sample of the ToyBox-intervention

In total, 2919 Belgian preschoolers’ parents/caregivers in the 27 kindergartens were contacted to participate and 2258 parents/caregivers (77.4 %) agreed to participate in the project. At baseline, 1199 preschoolers (53.1 %) got permission to wear an accelerometer. Due to absence of preschoolers on the day of device fitting, 1,105 preschoolers at baseline and 1123 preschoolers at follow-up were fitted with a device. A total of 859 preschoolers (29.5 % of contacted preschool children) had two weekdays and at least one weekend day with valid data at either baseline or follow-up. Preschoolers with valid accelerometer data had a mean age of 4.4 ± 0.6 years at baseline (54.4 % boys), with 330 preschoolers in the control kindergartens and 529 preschoolers in the intervention kindergartens (preschoolers in low SES kindergartens = 341, 39.7 %; medium SES kindergartens = 274, 31.9 %; high SES kindergartens = 244, 28.4 %). The mean accelerometer wearing time for baseline and follow-up was 11.8 ± 1.1 hours and 12.1 ± 3.3 hours respectively. The flow of participants through the study is illustrated in Fig. 2. The demographic characteristics can be found in Table 2.

**Table 2:** Descriptive characteristics of sedentary behaviour at baseline and follow-up in Belgian preschool children in intervention (*n* = 15) and control (*n* = 12) kindergartens

		BASELINE		FOLLOW-UP	
		Intervention	Control	Intervention	Control
Accelerometer	n	529	330	/	/
	Gender (% boys)	54.6	53.9	/	/
	Age (years)	4.4 ± 0.6	4.3 ± 0.6	/	/
	Weight status				
	- Underweight (%) - Normal weight (%) - Overweight (%) - Obese (%)	11.0	10.2	/	/
		79.4	78.9	/	/
		6.9	10.2	/	/
		2.8	0.7	/	/
	SB weekday (%/day)	44.8 ± 6.5	45.8 ± 6.8	44.5 ± 6.9	46.3 ± 10.5
	SB weekend day (%/day)	45.9 ± 10.0	46.7 ± 9.7	45.0 ± 10.4	45.8 ± 10.8
Questionnaire	n	961	754	/	/
	Gender (% boys)	52.7	52.4	/	/
	Age (years)	4.5 ± 0.5	4.4 ± 0.5	/	/
	Weight status				
	- Underweight (%) - Normal weight (%) - Overweight (%) - Obese (%)	12.3	9.7	/	/
		77.3	79.1	/	/
		8.2	9.7	/	/
		2.1	1.4	/	/
	TV week (min/day)	61.1 ± 43.1	67.9 ± 55.8	60.6 ± 44.3	68.6 ± 55.7
	TV weekend (min/day)	108.6 ± 72.5	117.7 ± 85.3	105.8 ± 69.8	119.1 ± 84.5
	PC week (min/day)	11.1 ± 25.5	11.8 ± 24.5	15.6 ± 30.6	16.4 ± 29.3
	PC weekend (min/day)	23.1 ± 42.9	23.5 ± 39.3	30.2 ± 44.7	32.3 ± 45.9
	Quiet play week (min/day)	71.7 ± 51.8	81.7 ± 66.1	70.7 ± 52.4	77.3 ± 61.8
	Quiet play weekend (min/day)	154.2 ± 95.2	158.9 ± 101.5	145.3 ± 113.6	151.6 ± 96.3

*SB* sedentary behaviour, *TV* television, *PC* personal computer

A total of 1715 parents/caregivers (58.9 %) (parents/caregivers of preschoolers in low SES kindergartens = 678, 39.5 %; medium SES kindergartens = 547, 31.9 %; high SES kindergartens = 490, 28.6 %) completed the questionnaire at either one of both measurement periods. Preschoolers whose parents/caregivers completed the questionnaire at either baseline or follow-up had a mean age of 4.4 ± 0.5 years at baseline, of which 52.5 % were boys. In total, 754 preschoolers (44.0 %) were located in the control kindergartens and 961 preschoolers (56.0 %) in the intervention kindergartens (Table 2).

### Effect of the ToyBox-intervention on preschoolers’ sedentary time (accelerometers)

No significant intervention effects on sedentary time were found for the total sample (all *p* > 0.05). After stratification, no significant intervention effects were found for objectively measured sedentary time in both boys and girls (all *p* > 0.05). Similarly, no intervention effects were found for objectively measured sedentary time in preschoolers from low and medium SES kindergartens (all *p* > 0.05). However, significant intervention effects were found for sedentary behaviour on weekdays and during school hours for preschoolers from high SES kindergartens. Preschool children from the intervention group had a decrease in sedentary behaviour on weekdays (-0.42 %) from baseline to follow-up, while preschoolers from the control group had an increase (+3.24 %) in sedentary behaviour on weekdays (β = -4.20; *p* = 0.03; d = 0.36). Furthermore, preschoolers from the intervention group had a decrease in sedentary behaviour during school hours (-2.00 %), compared to preschoolers from the control group in which sedentary behaviour increased (+0.47 %) from baseline to follow-up (β = -2.48; *p* = 0.04; d = 0.40). Results of the analyses can be found in Table 3 (total sample), Table 4 (stratified by gender) and Table 5 (stratified by SES).

**Table 3:** Time and interaction effects for sedentary behaviour outcomes in the total sample (adjusted for age and gender)

	Outcomes		Mean difference (follow-up - baseline)	Time		Time* Condition		
				β (SE)	95 % CI	β (SE)	95 % CI	*p*-value
Accelerometer^a^	SB weekday	I	+0.96 %	0.96 (0.62)	−0.26 to 2.19	−1.31 (0.78)	−2.84 to 0.21	0.09
		C	−0.35 %					
	SB weekend day	I	−0.86 %	−1.12 (0.75)	−2.59 to 0.35	0.26 (0.94)	−1.58 to 2.10	0.78
		C	−1.12 %					
	Total SB	I	−0.41 %	0.52 (0.56)	−0.57 to 1.62	−0.93 (0.70)	−2.30 to 0.43	0.18
		C	+0.52 %					
	SB school hours	I	−1.19 %	−0.18 (0.55)	−1.25 to 0.90	−1.01 (0.69)	−2.36 to 0.33	0.14
		C	−0.18 %					
	SB after school hours	I	−0.96 %	−1.37 (0.78)	−2.90 to 0.16	0.41 (0.97)	−1.49 to 2.32	0.67
		C	−1.37 %					
Questionnaire^b^	TV week	I	+0.13 min/day	0.73 (1.57)	−2.36 to 4.78	−0.59 (2.06)	−4.63 to 3.44	0.77
		C	+0.73 min/day					
	TV weekend	I	−2.61 min/day	1.90 (2.56)	−3.11 to 6.92	−4.51 (3.35)	−11.07 to 2.05	0.18
		C	+1.90 min/day					
	PC week	I	+4.79 min/day	5.83 (1.04)***	**3.79 to 7.86**	−1.04 (1.36)	−3.69 to 1.62	0.44
		C	+5.83 min/day					
	PC weekend	I	+8.12 min/day	11.17 (1.50)***	**8.23 to 14.11**	−3.05 (1.96)	−6.90 to 0.80	0.12
		C	+11.17 min/day					
	Quiet play week	I	−0.87 min/day	−4.44 (2.33)	−9.00 to 0.13	3.56 (3.05)	−2.41 to 9.53	0.24
		C	−4.44 min/day					
	Quiet play weekend	I	−9.19 min/day	−7.05 (4.59)	−16.05 to 1.95	−2.14 (5.99)	−13.89 to 9.60	0.72
		C	−7.05 min/day					

*SB* Sedentary Behaviour, *TV* Television, *PC* Personal Computer, *SE* Standard Error, 95 % *CI* 95 % Confidence Interval, *I* Intervention group, *C* Control group

*p < 0.05, **p < 0.01, ***p < 0.001

^a^n = 859 (I = 529, C = 330)

^b^n = 1715 (I = 961, C = 754)

**Table 4:** Time and interaction effects for sedentary behaviour outcomes in Belgian boys and girls (adjusted for age)

	Outcomes		Mean difference (follow-up - baseline)	Time		Time * Condition		
				β (SE)	95 % CI	β (SE)	95 % CI	*p*-value
BOYS^a^								
Accelerometer	SB weekday	I	+0.29 %	−0.29 (0.46)	−1.19 to 0.60	0.32 (0.78)	−1.21 to 1.85	0.68
		C	+0.03 %					
	SB weekend day	I	−0.50 %	−0.50 (0.71)	−1.88 to 0.89	-0.64 (1.21)	−3.00 to 1.73	0.60
		C	−1.13 %					
	Total SB	I	−0.23 %	−0.06 (0.60)	−1.22 to 1.11	-0.17 (0.74)	−1.61 to 1.27	0.82
		C	−0.06 %					
	SB school hours	I	−1.26 %	−0.85 (0.74)	−2.31 to 0.61	-0.41 (0.92)	−2.20 to 1.39	0.66
		C	−0.85 %					
	SB after school hours	I	−0.34 %	−2.90 (1.10)**	**−5.05 to -0.75**	2.56 (1.53)	−0.09 to 5.21	0.06
		C	−2.90 %					
Questionnaire	TV week	I	−0.22 min/day	1.31 (2.16)	−2.92 to 5.55	−1.53 (2.83)	−7.92 to 4.86	0.59
		C	+1.31 min/day					
	TV weekend	I	+0.29 min/day	−0.10 (3.44)	−6.85 to 6.64	0.39 (4.50)	−8.43 to 9.22	0.93
		C	−0.10 min/day					
	PC week	I	+4.24 min/day	6.96 (1.30)***	**4.42 to 9.51**	−2.72 (1.70)	−7.76 to 2.32	0.11
		C	+6.96 min/day					
	PC weekend	I	+8.26 min/day	13.69 (2.15)***	**9.48 to 17.90**	−5.42 (2.82)	−10.94 to 0.10	0.05
		C	+13.69 min/day					
	Quiet play week	I	−1.92 min/day	−4.87 (2.81)	−10.37 to 0.64	2.95 (3.68)	−4.26 to 10.15	0.42
		C	−4.87 min/day					
	Quiet play weekend	I	−5.24 min/day	−10.31 (6.85)	−23.72 to 3.11	5.07 (8.95)	−12.48 to 22.61	0.57
		C	−10.31 min/day					
GIRLS^b^								
Accelerometer	SB weekday	I	−0.42 %	1.98 (1.12)	−0.22 to 4.19	−2.41 (1.42)	−5.20 to 0.38	0.09
		C	+1.98 %					
	SB weekend day	I	−1.32 %	−1.10 (1.16)	−3.37 to 1.17	-0.21 (1.47)	−3.08 to 2.66	0.89
		C	−1.10 %					
	Total SB	I	−0.66 %	1.15 (0.98)	−0.77 to 3.08	-1.81 (1.25)	−4.25 to 0.63	0.15
		C	+1.15 %					
	SB school hours	I	−1.10 %	0.56 (0.81)	−1.03 to 2.15	-1.66 (1.03)	−3.68 to 0.36	0.11
		C	+0.56 %					
	SB after school hours	I	−1.74 %	0.30 (1.09)	−1.84 to 2.43	-2.03 (1.38)	−4.74 to 0.67	0.14
		C	+0.30 %					
Questionnaire	TV week	I	+0.52 min/day	0.06 (2.30)	−4.45 to 4.56	0.47 (3.00)	−5.41 to 6.34	0.88
		C	+0.06 min/day					
	TV weekend	I	−5.83 min/day	4.15 (3.81)	−3.32 to 11.62	−9.98 (4.98)*	**−19.74 to -0.23**	**0.04**
		C	+4.15 min/day					
	PC week	I	+5.40 min/day	4.56 (1.64)**	**1.34 to 7.79**	0.84 (2.15)	−3.37 to 5.05	0.70
		C	+4.56 min/day					
	PC weekend	I	+7.97 min/day	8.33 (2.08)***	**4.26 to 12.39**	−0.36 (2.71)	−5.68 to 4.95	0.89
		C	+8.33 min/day					
	Quiet play week	I	+0.28 min/day	−3.96 (3.80)	−11.41 to 3.49	4.24 (4.97)	−5.49 to 13.97	0.39
		C	−3.96 min/day					
	Quiet play weekend	I	−13.59 min/day	−3.34 (5.97)	−15.04 to 8.37	−10.25 (7.78)	−25.51 to 5.00	0.19
		C	−3.34 min/day					

*SB* Sedentary Behaviour, *TV* Television, *PC* Personal Computer, *SE* Standard Error, *95 % CI* 95 % Confidence Interval, *I* Intervention group, *C* Control group

*p < 0.05, **p < 0.01, ***p < 0.001

^a^accelerometer: n = 467 (I = 289; C = 178); questionnaire: n = 901 (I = 506; C = 395)

^b^accelerometer: n = 392 (I = 240; C = 152); questionnaire: n = 814 (I = 455; C = 359)

**Table 5:** Time and interaction effects for sedentary behaviour outcomes in preschoolers from low, medium and high SES kindergartens (adjusted for age and gender)

	Outcomes		Mean difference (follow-up - baseline)	Time		Time* Condition		
				β (SE)	95 % CI	β (SE)	95 % CI	p-value
LOW SES KINDERGARTENS^a^								
Accelerometer	SB weekday	I	−0.04 %	−0.04 (0.69)	−1.40 to 1.32	0.00 (0.92)	−1.80 to 1.80	0.99
		C	−0.04 %					
	SB weekend day	I	−1.51 %	−1.50 (1.07)	−3.59 to 0.60	-0.01 (1.42)	−2.79 to 2.77	0.99
		C	−1.50 %					
	Total SB	I	−0.47 %	−0.08 (0.62)	−1.31 to 1.14	-0.39 (0.83)	−2.01 to 1.23	0.64
		C	−0.08 %					
	SB school hours	I	−0.28 %	0.06 (0.85)	−1.61 to 1.72	−0.34 (1.13)	−2.55 to 1.88	0.77
		C	−0.06 %					
	SB after school hours	I	−2.07 %	−3.01 (1.12)**	**−5.20 to -0.81**	0.94 (1.48)	−1.97 to 3.84	0.53
		C	−3.01 %					
Questionnaire	TV week	I	+0.63 min/day	2.24 (2.05)	−1.77 to 6.25	1.61 (2.83)	−7.16 to 3.94	0.57
		C	+2.24 min/day					
	TV weekend	I	−3.55 min/day	−4.38 (3.78)	−11.79 to 3.03	0.83 (5.24)	−9.44 to 11.10	0.87
		C	−4.38 min/day					
	PC week	I	+4.01 min/day	5.83 (1.32)***	**3.25 to 8.41**	−1.81 (1.82)	−5.38 to 1.76	0.32
		C	+5.83 min/day					
	PC weekend	I	+6.06 min/day	12.49 (2.14)***	**8.29 to 16.69**	−6.43 (2.98)*	**−12.27 to -0.59**	**0.03**
		C	+12.49 min/day					
	Quiet play week	I	−2.76 min/day	−4.39 (3.24)	−10.74 to 1.95	1.64 (4.50)	−7.18 to 10.45	0.72
		C	−4.39 min/day					
	Quiet play weekend	I	−10.04 min/day	−6.65 (7.58)	−21.49 to 8.20	−3.39 (10.51)	−23.98 to 17.20	0.75
		C	−6.65 min/day					
MEDIUM SES KINDERGARTENS^b^								
Accelerometer	SB weekday	I	−0.01 %	0.24 (0.85)	−1.43 to 1.90	-0.25 (0.99)	−2.19 to 1.70	0.81
		C	+0.24 %					
	SB weekend day	I	−0.33 %	-0.14 (1.52)	−3.11 to 2.84	-0.19 (1.78)	−3.68 to 3.29	0.91
		C	+0.14 %					
	Total SB	I	+0.03 %	0.13 (0.83)	−1.50 to 1.76	-0.10 (0.97)	−2.01 to 1.81	0.92
		C	+0.13 %					
	SB school hours	I	−1.30 %	-1.21 (1.01)	−3.19 to 0.78	-0.10 (1.19)	−2.42 to 2.23	0.94
		C	−1.21 %					
	SB after school hours	I	+0.16 %	−0.12 (1.30)	−2.67 to 2.43	0.28 (1.52)	−2.71 to 3.26	0.86
		C	−0.12 %					
Questionnaire	TV week	I	+1.66 min/day	1.95 (3.49)	−4.89 to 8.78	−0.29 (4.25)	−8.61 to 8.04	0.94
		C	+1.95 min/day					
	TV weekend	I	−0.98 min/day	7.79 (5.37)	−2.74 to 18.31	−8.76 (1.79)	−21.62 to 4.09	0.18
		C	+7.79 min/day					
	PC week	I	+6.25 min/day	5.38 (2.74)*	**0.01 to 10.75**	0.86 (3.34)	−5.68 to 7.41	0.80
		C	+5.38 min/day					
	PC weekend	I	+10.45 min/day	7.39 (3.47)*	**0.58 to 14.19**	3.07 (4.23)	−5.23 to 11.36	0.47
		C	+7.38 min/day					
	Quiet play week	I	+3.08 min/day	−7.67 (4.50)	−16.49 to 1.15	10.75 (5.49)	−0.01 to 25.51	0.05
		C	−7.67 min/day					
	Quiet play weekend	I	−0.51 min/day	−12.44 (7.93)	−27.98 to 3.09	11.93 (9.66)	−6.99 to 30.86	0.22
		C	−12.44 min/day					
HIGH SES KINDERGARTENS^c^								
Accelerometer	SB weekday	I	−0.42 %	3.24 (1.61)*	**0.08 to 6.40**	−4.20 (1.93)*	**−7.98 to -0.43**	**0.03**
		C	+3.24 %					
	SB weekend day	I	−1.54 %	−1.49 (1.35)	−4.13 to 1.15	−0.05 (1.61)	−3.21 to 3.11	0.98
		C	−1.49 %					
	Total SB	I	−1.14 %	1.85 (1.40)	−0.89 to 4.60	−2.99 (1.68)	−6.28 to 0.30	0.08
		C	+1.85 %					
	SB school hours	I	−2.00 %	0.48 (1.03)	−1.54 to 2.49	-2.48 (1.23)*	**−4.88 to -0.07**	**0.04**
		C	+0.47 %					
	SB after school hours	I	−1.51 %	−0.06 (1.64)	−3.27 to 3.16	-1.45 (1.96)	−5.30 to 2.39	0.46
		C	−0.06 %					
Questionnaire	TV week	I	−2.25 min/day	−2.62 (3.06)	−8.62 to 3.38	0.37 (4.00)	−7.48 to 8.22	0.93
		C	−2.62 min/day					
	TV weekend	I	−3.35 min/day	7.04 (4.50)	−1.79 to 15.87	−10.39 (5.88)	−21.92 to 1.14	0.08
		C	+7.04 min/day					
	PC week	I	+4.07 min/day	6.19 (1.58)***	**3.10 to 9.27**	−2.11 (2.06)	−6.15 to 1.93	0.31
		C	+6.19 min/day					
	PC weekend	I	+7.98 min/day	12.04 (2.47)***	**7.20 to 16.87**	−4.05 (3.23)	−10.39 to 2.28	0.21
		C	+12.04 min/day					
	Quiet play week	I	−3.15 min/day	−1.98 (4.79)	−11.37 to 7.42	−1.18 (6.28)	−13.49 to 11.13	0.85
		C	−1.98 min/day					
	Quiet play weekend	I	−18.28 min/day	−3.48 (8.10)	−19.35 to 12.39	−14.81 (10.57)	−35.52 to 5.91	0.16
		C	−3.48 min/day					

*SB* Sedentary Behaviour, *TV* Television, *PC* Personal Computer, *SE* Standard Error, *95 % CI* 95 % Confidence Interval, *I* Intervention group, *C* Control group, *SES* Socio-economic status

*p < 0.05, **p < 0.01, ***p < 0.001

^a^accelerometer: n = 341 (I = 190; C = 151); questionnaire: n = 678 (I = 338; C = 340)

^b^accelerometer: n = 274 (I = 173; C = 101); questionnaire: n = 547 (I = 350; C = 197)

^c^accelerometer: n = 244 (I = 166; C = 78); questionnaire: n = 490 (I = 273; C = 217)

### Effect of the ToyBox-intervention on preschoolers’ screen time and quiet play (Primary Caregivers’ Questionnaire)

In the total sample, no significant intervention effects were found for subjectively measured sedentary behaviour (all *p* > 0.05). After stratifying the data according to gender, a significant intervention effect was found for preschool girls for TV viewing on weekend days (β = -9.98; *p* = 0.04; d = 0.23). More specifically, preschool girls from the intervention group had a decrease in TV viewing on weekend days going from baseline to follow-up (-5.83 min/day), compared to the control group in which preschool girls had an increase in TV viewing on weekend days (+4.15 min/day). No significant intervention effects on subjectively measured sedentary behaviour were found for preschool boys (all at *p* > 0.05).

Furthermore, a significant intervention effect was found for computer use on weekend days in preschoolers from low SES kindergartens (β = -6.43; *p* = 0.03; d = 0.11). Preschoolers from the intervention group had a smaller increase in computer use on weekend days (+6.06 min/day) going from baseline to follow-up compared to preschoolers from the control group in which a steeper increase was found (+12.49 min/day). No significant intervention effects on subjectively measured sedentary behaviour were found for preschoolers from medium and high SES kindergartens (all at *p* > 0.05).

### Effect of the intervention on preschoolers with the highest levels of sedentary time at baseline

After stratifying the data to examine intervention effects in preschoolers with the highest levels of sedentary time at baseline, significant intervention effects were found for sedentary behaviour on weekdays and total sedentary behaviour (Table 6). Preschoolers from the intervention group had a decrease (β = -4.27; *p* = 0.04; d = 0.33) in sedentary behaviour on weekdays (-3.47 %) going from baseline to follow-up compared to preschoolers from the control group in which an increase was found (+0.80 %). Furthermore, preschoolers from the intervention group had a steeper decrease (β = -3.76; *p* = 0.04; d = 0.35) in total sedentary behaviour (-4.17 %) going from baseline to follow-up compared to preschoolers from the control group in which a smaller decrease was found (-0.41 %).

**Table 6:** Time and interaction effects for sedentary behaviour outcomes in the high risk group^a^ (adjusted for age and gender)

	Outcomes		Mean difference (follow-up - baseline)	Time		Time* Condition		
				β (SE)	95 % CI	β (SE)	95 % CI	*p*-value
Accelerometer	SB weekday	I	−3.47 %	0.80 (1.68)	−2.49 to 4.09	−4.27 (2.10)*	**−8.39 to -0.15**	**0.04**
		C	+0.80 %					
	SB weekend day	I	−6.24 %	−4.90 (1.56)***	**−7.97 to -1.84**	−1.34 (1.96)	−5.18 to 2.50	0.49
		C	−4.90 %					
	Total SB	I	−4.17 %	−0.41 (1.47)	−3.29 to 2.48	−3.76 (1.85)*	**−7.38 to -0.15**	**0.04**
		C	−0.41 %					
	SB school hours	I	−3.96 %	−1.22 (1.18)	−3.53 to 1.09	−2.74 (1.48)	−5.64 to 0.16	0.06
		C	−1.22 %					
	SB after school hours	I	−5.86 %	−3.38 (1.56)*	**−6.43 to -0.34**	−2.48 (1.95)	−6.30 to 1.34	0.20
		C	−3.38 %					

*SB* Sedentary Behaviour, *SE* Standard Error, *95 % CI* 95 % Confidence Interval, *I* Intervention group, *C* Control group

*p < 0.05, **p < 0.01, ***p < 0.001

^a^Preschoolers with high levels of sedentary time at pre-test (>P70; 70^th^ percentile of the total sedentary time at baseline), n = 256; I = 150, C = 106

## Discussion

The present study investigated the effect of the sedentary behaviour module of the ToyBox-intervention on Belgian preschoolers’ sedentary behaviour. In contrast with other studies [29, 67], this intervention study not only focused on TV viewing time or screen viewing time, given that a decrease of total sedentary time and other sedentary activities were targeted as well. In the total sample, no intervention effect was found on preschoolers’ objectively and subjectively measured total sedentary time. However, some favourable effects were found in different sub-groups, namely in girls, high SES kindergartens and preschoolers with the highest levels of sedentary behaviour at baseline. However, the effect sizes are small, which means that these results should be interpreted with caution and that the biological relevance might be questioned.

In the current study, only in preschool girls an intervention effect was found for TV viewing on weekend days. This was surprising, as previous studies have shown that preschool boys spend more time in screen-based activities compared to girls [68, 69], and for that reason, one might have expected significant effects in preschool boys. Although the same intervention was applied for boys and girls, this result might suggest that the intervention materials were better suited for preschool girls compared to preschool boys.

The development of the ToyBox-intervention was based on the socio-ecological model and targeted kindergartens with different SES levels. Therefore, potential differences in intervention effects for low, medium and high SES kindergarten levels were investigated. Only in high SES kindergartens, an intervention effect was found for objectively measured sedentary time, namely during weekdays and during school hours. There was a 0.4 % decrease in sedentary time during weekdays in preschoolers from the intervention group compared to a 3.2 % increase in preschoolers from the control group. Taking an accelerometer wearing time of ten hours per day into account, this 2.8 % decrease in sedentary time corresponds to 2.4 minutes.

In low SES kindergartens, an effect of the intervention was found for computer use during weekend days. In both intervention and control groups, computer use on weekend days increased, but the ToyBox-intervention was effective in slowing down this increase in preschoolers from low SES kindergartens. This result is promising, although one should keep in mind that computer use was proxy-reported by the parents and thus not objectively measured, which might have introduced bias.

Kindergarten SES was based on the SES of the municipality, which means that low or high SES kindergartens were located in low or high SES neighbourhoods. Children from low SES neighbourhoods have less access to a private garden at home, a park or suitable nearby nature [70]. It might be possible that these preschool children spend more time on their computer, and less time outside. Thus, a possible explanation might be that preschoolers’ parents in these low SES neighbourhoods are aware of this problem, and that they were motivated to put the tips and tricks on computer use (included on the intervention materials) into practice which might have caused the smaller increase in computer use on weekend days. However, another explanation might be that the parents from preschoolers from these low SES kindergartens were more likely to be subject to reporting bias. Either way, the results seem to suggest that a passive parent approach in preschool children from low SES neighbourhoods might work and might induce a smaller increase in computer use.

Finally, the ToyBox-intervention was effective in decreasing the sedentary time during weekdays and for total sedentary behaviour in preschoolers from the high risk group, which means that the intervention was effective in preschoolers with the highest levels of objectively measured sedentary time at baseline. These results show that the ToyBox-intervention worked within the group of preschoolers that needed the intervention the most.

The ToyBox-intervention focussed on four different behaviours (i.e., water consumption, healthy snacking, physical activity and sedentary behaviour) during one school year. This means that only a limited period of time was available to implement the sedentary behaviour module. However, teachers were encouraged to continue the implementation of several parts of the sedentary behaviour intervention throughout the school year. Because sedentary behaviour has a strong habitual component, this behaviour might be difficult to change during a relative short intervention period. A recent meta-analysis indicated that interventions to reduce sedentary time that lasted less than 4 months only had small intervention effects [22], so future interventions might consider implementing interventions for a longer period of time to enhance the present intervention effects [23]. Another possible reason for the lack of more intervention effects in the current study might be the fact that sedentary behaviour is a new concept for most kindergarten teachers. It is possible that they were not convinced of the possible health effects and that this is a reason for not implementing the intervention as intended or for struggling with the intervention content. However, this is only a hypothesis which should be further clarified through process evaluation data. Furthermore, no intervention effects were found for time spent in quiet play. It is possible that parents as well as teachers did not find it necessary to decrease preschoolers’ time spent in quiet play as they perceived it essential that preschoolers perform quiet play activities (e.g., puzzling, playing with blocks, colouring, etc.) for the development of their cognitive development and their fine movement skills (i.e. productive sedentary behaviour) [19]. These activities are indeed essential for preschoolers’ development, but further research should focus on increasing parents’ and teachers’ awareness on how prolonged periods in quiet play can be interrupted or can be performed while standing. For example, colouring or painting can easily be done while standing up instead of sitting down. This was also included in the Intervention Mapping approach [32], in which performance objectives for preschool children, parents and teachers were formulated regarding decreasing the total sitting time and switching from sitting down to standing up for different activities. This is why the intervention material mainly focused on interrupting prolonged periods of sitting time and screen viewing time. However, it might have been better to include literal messages regarding quiet play instead of focussing on interrupting sitting time. Future interventions could try to provide clear and simple messages for both parents and teachers regarding preschoolers’ quiet play. In addition, it might be possible that the accelerometers did not capture these small breaks in sedentary time, as accelerometers cannot distinguish different postures such as sitting, standing up or lying down [71, 72]. Future studies might benefit from using inclinometers – such as the ActivPal – to measure the breaks in sedentary time during the day [73].

In the ToyBox-intervention, parents/caregivers were involved in a rather passive way. They were provided with educational materials, including tips and tricks, and suggestions for parent-child activities to affect a decrease in sedentary time over time [74]. To achieve a decrease of time spent in different sedentary activities, a more intense delivery style of the intervention and the inclusion of parents as direct intervention targets might be necessary [75]. This might increase parental motivation to focus on decreasing their child’s sedentary behaviour at home. Finally, analysing the detailed process-evaluation about the implementation of the intervention by teachers and parents/caregivers will provide insight in how and which activities teachers implemented at kindergarten, how the educational materials were perceived by the parents/caregivers and if the execution of parts of the sedentary behaviour module were continued throughout the school year.

Study limitations include the reliance on parental reports of their children’s screen viewing time and time spent in quiet play and the large drop-out of preschoolers due to the lack of valid accelerometer data. In addition, using proxy-reports to assess preschoolers’ time spent in sedentary activities might induce bias as well, as preschoolers’ parents are unable to constantly monitor their child’s behaviour [76]. For this reason, the generalizability of the findings might be questioned. Furthermore, the lack of a follow-up study and the lack of including process evaluation data might be considered as a limitation. Finally, it should be acknowledged that the timing of the baseline and follow-up measurements was not ideal. It is possible that between the baseline measurements and the actual start of the implementation of the intervention activities (e.g., theme week where kindergarten teachers try to increase preschoolers’ physical activity levels) were performed at kindergarten or at home that could potentially influence preschoolers’ sedentary behaviour. Furthermore, some of the follow-up measurements were also performed before the actual end of the intervention period. The execution of the first two parts of the classroom activity guide continued until the end of the school year and potential intervention effects that were present at the end of the school year could therefore not be detected.

Important strengths of this study were the assessment of preschoolers’ sedentary behaviour with both objective and subjective measurement tools in a large sample of 4- to 6-year-old Belgian preschool children, which enabled the measurement of intensity, frequency, duration, as well as type and context of preschool children’s sedentary behaviour [77]. Another strength was the use of a randomized controlled cluster intervention with a pre-test post-test design to examine intervention effects.

## Conclusions

The ToyBox-intervention had no effect on objectively and subjectively measured sedentary time in the total sample. However, some small effects were found in specific sub-groups (preschool girls, preschoolers from low and high SES kindergartens, preschoolers with higher levels of sedentary time at baseline). Because the ToyBox-intervention was a multi-component intervention implemented at kindergarten, only a limited period of time was available to implement the sedentary behaviour component and parents/caregivers were included in a rather passive way. To enlarge effects of interventions focusing on decreasing preschoolers’ sedentary behaviour, longer intervention periods focusing on sedentary behaviour and a more active involvement of parents might be necessary.
